# Supplementary material for: The first moment of income density functions and estimation of single-parametric Lorenz curves
Source: PLoS One. 2022 Jun 24;17(6):e0267828. doi: 10.1371/journal.pone.0267828 (PMC9231794; doi:10.1371/journal.pone.0267828)
Supplement: S1 Appendix — (DOCX) [file pone.0267828.s001.docx]

**APPENDIX A**

**Fig. 16** Illustration of the size of MDC by Chotikapanich and RGKO LCs

The tangential line of the RGKO LC is as follows:

$$y=p+MIS-MPS=p+2^{1-1/\alpha}-1$$

The tangential line of the Chotikapanich LC is as follows:

$$y=p+\frac{1}{\sigma}-\frac{1}{e^{\sigma}-1}-\frac{1}{\sigma}ln\frac{e^{\sigma}-1}{\sigma}$$
