# Supplementary figures and images for: The first moment of income density functions and estimation of single-parametric Lorenz curves
Source: PLoS One. 2022 Jun 24;17(6):e0267828. doi: 10.1371/journal.pone.0267828 (PMC9231794; doi:10.1371/journal.pone.0267828)

**APPENDIX B**


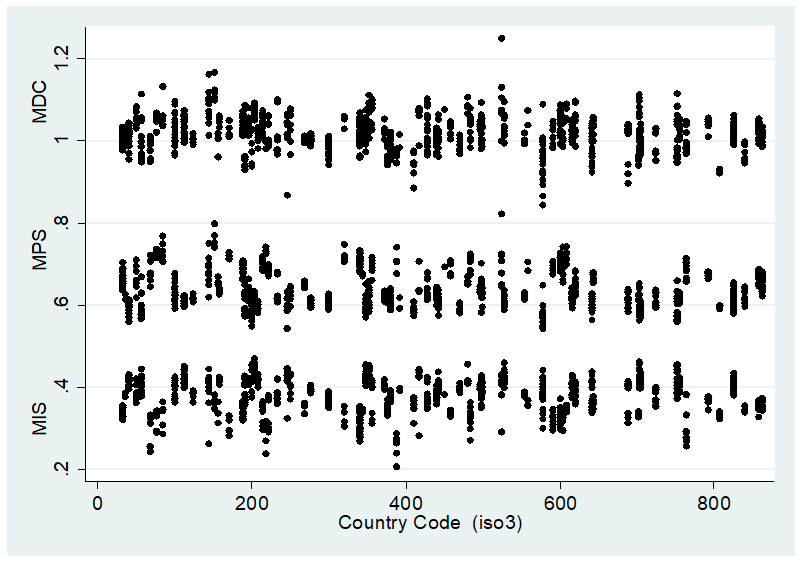


**Fig. 17** Observations of the first moments by country

Supplement: S2 Appendix — (DOCX) [file pone.0267828.s002.docx]

**APPENDIX C**

**Fig. 18** 1000 random LCs within and across critical boundaries

**
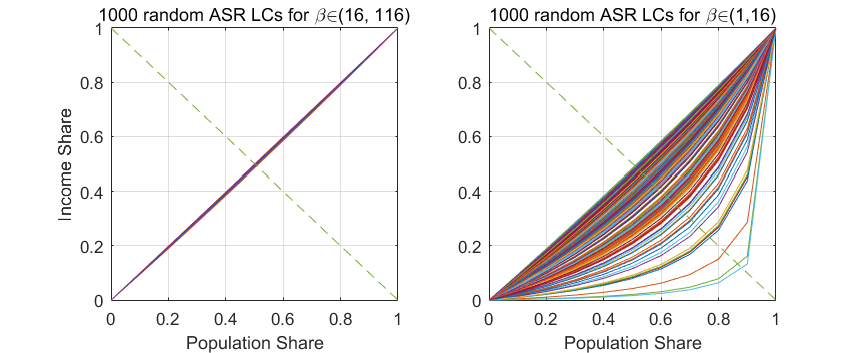
**

**
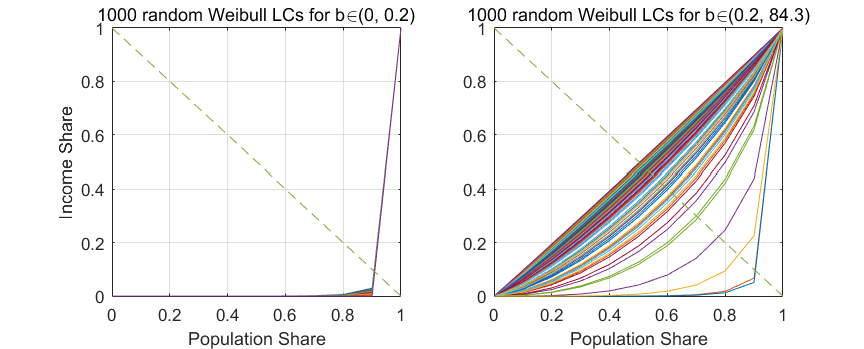
**

**
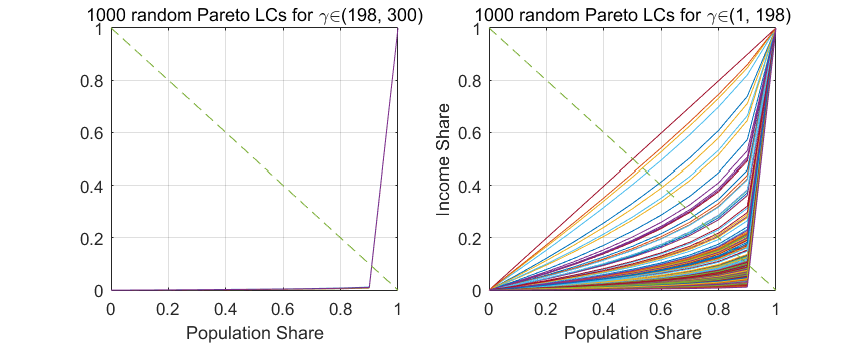
**

**
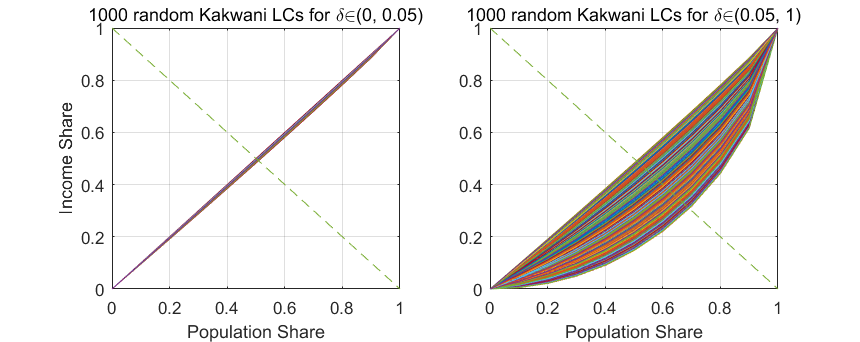
**

**
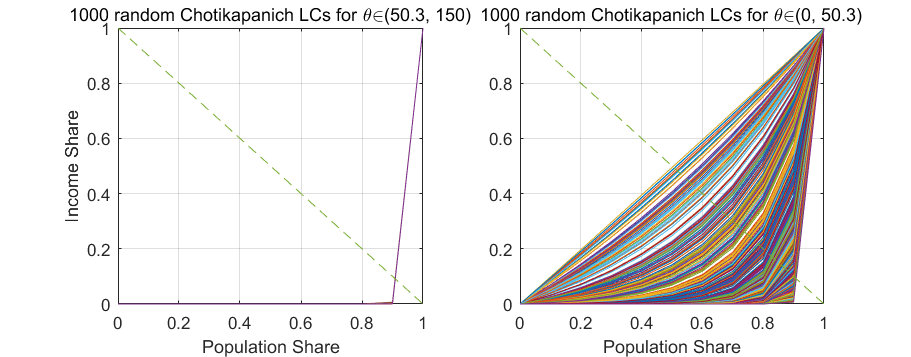
**

**
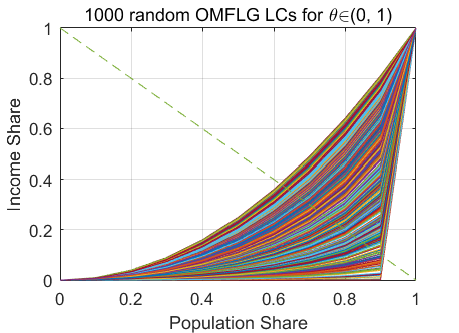
**

Supplement: S3 Appendix — (DOCX) [file pone.0267828.s003.docx]
