# Supplementary material for: The first moment of income density functions and estimation of single-parametric Lorenz curves
Source: PLoS One. 2022 Jun 24;17(6):e0267828. doi: 10.1371/journal.pone.0267828 (PMC9231794; doi:10.1371/journal.pone.0267828)
Supplement: S4 Appendix — (DOCX) [file pone.0267828.s004.docx]

**APPENDIX D**

**Proof for Proposition 1**

We use the following equation for the LC (Gastwirth, 1971):

$$L\left( p \right)=\frac{1}{\mu}\int_{0}^{p} F^{-1}\left( t \right)dt, \mu=\int_{0}^{\infty} xdF\left( x \right)$$

The first derivative of the LC is

$$L^{'}\left( p \right)=\frac{F^{-1}\left( p \right)}{\mu}$$

Employing the definition of the MPS, $MPS=F\left( \mu\right),$ we obtain

$$L^{'}\left( MPS \right)=\frac{F^{-1}\left( MPS \right)}{\mu}=1, MIS=L\left( MPS \right) ▯$$

**Proof for Proposition 2.**

Theorem 1 in Krause (2014) shows the following equation for the income density $f\left( x \right)$ and LC $L\left( p \right)$:

$$f\left( x \right)=\frac{1}{\mu L''\left( F\left( x \right) \right)}$$

Since $f\left( x \right)$ is differentiable and $L\left( p \right)$ is $C^{3}$, there exists a real solution $p_{m}$ to the equation $L^{'''}\left( p_{m} \right)=0.$ Therefore, we have the following result:

$$f^{'}\left( x_{m} \right)=\frac{-L^{'''}\left( p_{m} \right)}{\mu\left[ L^{''}\left( F\left( x_{m} \right) \right) \right]^{2}}=0$$

Considering the inequality conditions, $L^{'''}\left( p_{m}+ \right)>0, L^{'''}\left( p_{m}- \right)<0,$ we obtain

$$f^{'}\left( x_{m}+ \right)<0, f^{'}\left( x_{m}- \right)>0,$$

We thus conclude that $x_{m}$ is a mode of $f\left( x \right).$ $▯$
