# Supplementary material for: The first moment of income density functions and estimation of single-parametric Lorenz curves
Source: PLoS One. 2022 Jun 24;17(6):e0267828. doi: 10.1371/journal.pone.0267828 (PMC9231794; doi:10.1371/journal.pone.0267828)
Supplement: S5 Appendix — (DOCX) [file pone.0267828.s005.docx]

**APPENDIX E**

Estimation Results

**Table 9** Estimation Results of the Eight Functions in Fitting the Grouped Income Data

| ID | Year | xrgko | **RGKO** | xkak | Kakwani | xweibl | Weibull | xomflg | OMFLG |
| --- | --- | --- | --- | --- | --- | --- | --- | --- | --- |
| ISR | 2007 | 0.6670 | **0.0157** | 0.7696 | 0.0391 | 1.2995 | 0.0389 | 0.7282 | 0.0564 |
| USA | 1994 | 0.6816 | **0.0191** | 0.7295 | 0.0496 | 1.3937 | 0.0333 | 0.7816 | 0.0609 |
| HRV | 2010 | 0.7338 | **0.0171** | 0.5875 | 0.0622 | 1.8182 | 0.0283 | 1.0040 | 0.0895 |
| ISR | 2005 | 0.6624 | **0.0133** | 0.7822 | 0.0346 | 1.2713 | 0.0418 | 0.7116 | 0.0562 |
| GRC | 2015 | 0.7164 | **0.0142** | 0.6336 | 0.0556 | 1.6607 | 0.0364 | 0.9210 | 0.0862 |
| SGP | 2011 | 0.6749 | **0.0147** | 0.7476 | 0.0430 | 1.3502 | 0.0390 | 0.7560 | 0.0616 |
| BGR | 2006 | 0.7363 | **0.0059** | 0.5800 | 0.0514 | 1.8461 | 0.0394 | 1.0122 | 0.1018 |
| LVA | 2008 | 0.6893 | **0.0080** | 0.7064 | 0.0401 | 1.4485 | 0.0448 | 0.8064 | 0.0770 |
| LTU | 2011 | 0.7238 | **0.0139** | 0.6137 | 0.0570 | 1.7250 | 0.0329 | 0.9545 | 0.0881 |
| GRC | 2014 | 0.7141 | **0.0089** | 0.6392 | 0.0498 | 1.6418 | 0.0407 | 0.9089 | 0.0893 |
| ITA | 2014 | 0.7300 | **0.0129** | 0.5971 | 0.0564 | 1.7846 | 0.0370 | 0.9838 | 0.0951 |
| FRA | 1981 | 0.7339 | **0.0097** | 0.5864 | 0.0518 | 1.8205 | 0.0382 | 0.9995 | 0.0991 |
| LVA | 2009 | 0.6922 | **0.0099** | 0.6986 | 0.0438 | 1.4701 | 0.0425 | 0.8189 | 0.0766 |
| EST | 2015 | 0.7109 | **0.0100** | 0.6477 | 0.0484 | 1.6138 | 0.0399 | 0.8943 | 0.0867 |
| BEL | 1995 | 0.7371 | **0.0231** | 0.5770 | 0.0437 | 1.8582 | 0.0575 | 1.0110 | 0.1178 |
| ISL | 2005 | 0.7839 | **0.0192** | 0.4592 | 0.0342 | 2.4384 | 0.0556 | 1.2892 | 0.1426 |
| PRY | 2014 | 0.5949 | **0.0388** | 0.9622 | 0.0562 | 0.9372 | 0.0987 | 0.5032 | 0.0657 |
| BLR | 1998 | 0.7851 | **0.0185** | 0.4561 | 0.0317 | 2.4560 | 0.0554 | 1.2950 | 0.1434 |
| PRY | 2007 | 0.5917 | **0.0375** | 0.9723 | 0.0570 | 0.9241 | 0.0974 | 0.4956 | 0.0621 |
| DOM | 2004 | 0.6036 | **0.0381** | 0.9373 | 0.0494 | 0.9735 | 0.0973 | 0.5253 | 0.0706 |
| CZE | 2010 | 0.7832 | **0.0192** | 0.4609 | 0.0323 | 2.4273 | 0.0563 | 1.2822 | 0.1432 |
| CZE | 2008 | 0.7846 | **0.0189** | 0.4574 | 0.0321 | 2.4485 | 0.0558 | 1.2920 | 0.1435 |
| PHL | 1991 | 0.5957 | **0.0480** | 0.9566 | 0.0646 | 0.9398 | 0.1073 | 0.5032 | 0.0756 |
| MEX | 2014 | 0.6100 | **0.0430** | 0.9178 | 0.0498 | 1.0023 | 0.1017 | 0.5412 | 0.0789 |
| CZE | 1992 | 0.8168 | **0.0186** | 0.3807 | 0.0311 | 3.0236 | 0.0499 | 1.5673 | 0.1494 |
| PER | 1994 | 0.5727 | **0.0425** | 1.0000 | 0.0805 | 0.8495 | 0.1029 | 0.4489 | 0.0564 |
| PHL | 1994 | 0.5791 | **0.0554** | 0.9996 | 0.0846 | 0.8736 | 0.1154 | 0.4616 | 0.0731 |
| CHL | 2011 | 0.5922 | **0.0510** | 0.9657 | 0.0702 | 0.9253 | 0.1104 | 0.4939 | 0.0765 |
| CHL | 1995 | 0.5714 | **0.0528** | 1.0000 | 0.0914 | 0.8443 | 0.1130 | 0.4440 | 0.0661 |
| CHL | 2013 | 0.5953 | **0.0502** | 0.9573 | 0.0671 | 0.9382 | 0.1097 | 0.5017 | 0.0775 |
| The first three columns are the first moments. The variable affixed with x denotes the parameter of the function in the next column; and the variable named by a function is the standardized SSE_LC. This table contains the first 15 and last 15 observations of the 666 observations that are best fitted by the RGKO LC and are ordered by the size of the MDC. | | | | | | | | | |

**Table 9** Continued

| xpart | Pareto | xasr | ASR | xchoti | Chotikapanich | xgup | Gupta |
| --- | --- | --- | --- | --- | --- | --- | --- |
| 2.2815 | 0.1282 | 1.3945 | 0.0352 | 2.7055 | 0.0488 | 6.0871 | 0.0566 |
| 2.1734 | 0.1283 | 1.4370 | 0.0377 | 2.5325 | 0.0435 | 5.2883 | 0.0498 |
| 1.8529 | 0.1098 | 1.6321 | 0.0343 | 1.9731 | 0.0337 | 3.4269 | 0.0371 |
| 2.3185 | 0.1271 | 1.3818 | 0.0344 | 2.7620 | 0.0519 | 6.3775 | 0.0602 |
| 1.9529 | 0.1117 | 1.5582 | 0.0399 | 2.1462 | 0.0448 | 3.9033 | 0.0491 |
| 2.2236 | 0.1258 | 1.4170 | 0.0398 | 2.6083 | 0.0507 | 5.6214 | 0.0574 |
| 1.8433 | 0.0976 | 1.6435 | 0.0323 | 1.9436 | 0.0405 | 3.3502 | 0.0453 |
| 2.1246 | 0.1136 | 1.4609 | 0.0348 | 2.4383 | 0.0506 | 4.9005 | 0.0577 |
| 1.9086 | 0.1099 | 1.5882 | 0.0325 | 2.0720 | 0.0365 | 3.6900 | 0.0413 |
| 1.9676 | 0.1075 | 1.5491 | 0.0370 | 2.1684 | 0.0463 | 3.9690 | 0.0516 |
| 1.8764 | 0.1050 | 1.6154 | 0.0395 | 2.0063 | 0.0441 | 3.5112 | 0.0479 |
| 1.8548 | 0.0998 | 1.6318 | 0.0253 | 1.9698 | 0.0348 | 3.4164 | 0.0406 |
| 2.1042 | 0.1150 | 1.4708 | 0.0372 | 2.4051 | 0.0497 | 4.7735 | 0.0562 |
| 1.9852 | 0.1088 | 1.5364 | 0.0292 | 2.2043 | 0.0409 | 4.0801 | 0.0474 |
| 1.8441 | 0.0862 | 1.6471 | 0.0426 | 1.9299 | 0.0563 | 3.3133 | 0.0616 |
| 1.6254 | 0.0591 | 1.9164 | 0.0387 | 1.5005 | 0.0502 | 2.4375 | 0.0543 |
| 2.9971 | 0.0924 | 1.2352 | 0.0593 | 3.7014 | 0.1237 | 14.4475 | 0.1345 |
| 1.6198 | 0.0572 | 1.9240 | 0.0337 | 1.4913 | 0.0468 | 2.4218 | 0.0514 |
| 3.0370 | 0.0954 | 1.2297 | 0.0601 | 3.7561 | 0.1241 | 15.1878 | 0.1347 |
| 2.8937 | 0.0906 | 1.2504 | 0.0549 | 3.5624 | 0.1185 | 12.7381 | 0.1296 |
| 1.6285 | 0.0580 | 1.9107 | 0.0368 | 1.5071 | 0.0493 | 2.4485 | 0.0538 |
| 1.6223 | 0.0574 | 1.9205 | 0.0355 | 1.4953 | 0.0482 | 2.4285 | 0.0527 |
| 2.9879 | 0.0819 | 1.2362 | 0.0607 | 3.6856 | 0.1298 | 14.2470 | 0.1411 |
| 2.8234 | 0.0855 | 1.2622 | 0.0597 | 3.4567 | 0.1211 | 11.5818 | 0.1321 |
| 1.4946 | 0.0465 | 2.1928 | 0.0329 | 1.2300 | 0.0414 | 2.0329 | 0.0447 |
| 3.2893 | 0.0915 | 1.1995 | 0.0597 | 4.0968 | 0.1342 | 20.8619 | 0.1451 |
| 3.2015 | 0.0771 | 1.2090 | 0.0669 | 3.9699 | 0.1431 | 18.5319 | 0.1544 |
| 3.0313 | 0.0798 | 1.2301 | 0.0627 | 3.7434 | 0.1339 | 15.0198 | 0.1453 |
| 3.3091 | 0.0808 | 1.1973 | 0.0647 | 4.1172 | 0.1435 | 21.2814 | 0.1546 |
| 2.9941 | 0.0802 | 1.2354 | 0.0631 | 3.6911 | 0.1326 | 14.3169 | 0.1439 |

**Table 10** Estimation Results of the Eight Functions in Capturing the First Moments

| ID | Year | **RGKO** | xrgko | Kakwani | xkak | Choti | xchoti | ASR | xasr |
| --- | --- | --- | --- | --- | --- | --- | --- | --- | --- |
| ISR | 2007 | **0.0300** | 0.6670 | 0.0562 | 0.7696 | 0.0388 | 2.7055 | 0.0309 | 1.3945 |
| USA | 1994 | **0.0297** | 0.6816 | 0.0669 | 0.7295 | 0.0360 | 2.5325 | 0.0306 | 1.4370 |
| HRV | 2010 | **0.0269** | 0.7338 | 0.1027 | 0.5875 | 0.0273 | 1.9731 | 0.0278 | 1.6321 |
| ISR | 2005 | **0.0262** | 0.6624 | 0.0490 | 0.7822 | 0.0434 | 2.7620 | 0.0272 | 1.3818 |
| GRC | 2015 | **0.0255** | 0.7164 | 0.0888 | 0.6336 | 0.0327 | 2.1462 | 0.0269 | 1.5582 |
| SGP | 2011 | **0.0252** | 0.6749 | 0.0575 | 0.7476 | 0.0419 | 2.6083 | 0.0266 | 1.4170 |
| BGR | 2006 | **0.0247** | 0.7363 | 0.1025 | 0.5800 | 0.0288 | 1.9436 | 0.0257 | 1.6435 |
| LVA | 2008 | **0.0245** | 0.6893 | 0.0679 | 0.7064 | 0.0393 | 2.4383 | 0.0258 | 1.4609 |
| LTU | 2011 | **0.0243** | 0.7238 | 0.0929 | 0.6137 | 0.0319 | 2.0720 | 0.0251 | 1.5882 |
| GRC | 2014 | **0.0243** | 0.7141 | 0.0860 | 0.6392 | 0.0345 | 2.1684 | 0.0259 | 1.5491 |
| ITA | 2014 | **0.0241** | 0.7300 | 0.0974 | 0.5971 | 0.0310 | 2.0063 | 0.0255 | 1.6154 |
| FRA | 1981 | **0.0237** | 0.7339 | 0.0997 | 0.5864 | 0.0299 | 1.9698 | 0.0241 | 1.6318 |
| LVA | 2009 | **0.0228** | 0.6922 | 0.0684 | 0.6986 | 0.0405 | 2.4051 | 0.0244 | 1.4708 |
| EST | 2015 | **0.0228** | 0.7109 | 0.0822 | 0.6477 | 0.0360 | 2.2043 | 0.0236 | 1.5364 |
| BEL | 1995 | **0.0234** | 0.7371 | 0.1017 | 0.5770 | 0.0321 | 1.9299 | 0.0261 | 1.6471 |
| ISL | 2005 | **0.0507** | 0.7839 | 0.0601 | 0.4592 | 0.0929 | 1.5005 | 0.0513 | 1.9164 |
| PRY | 2014 | **0.0511** | 0.5949 | 0.0772 | 0.9622 | 0.1336 | 3.7014 | 0.0530 | 1.2352 |
| BLR | 1998 | **0.0519** | 0.7851 | 0.0597 | 0.4561 | 0.0937 | 1.4913 | 0.0523 | 1.9240 |
| PRY | 2007 | **0.0521** | 0.5917 | 0.0809 | 0.9723 | 0.1353 | 3.7561 | 0.0540 | 1.2297 |
| DOM | 2004 | **0.0522** | 0.6036 | 0.0717 | 0.9373 | 0.1330 | 3.5624 | 0.0536 | 1.2504 |
| CZE | 2010 | **0.0538** | 0.7832 | 0.0566 | 0.4609 | 0.0960 | 1.5071 | 0.0542 | 1.9107 |
| CZE | 2008 | **0.0543** | 0.7846 | 0.0570 | 0.4574 | 0.0962 | 1.4953 | 0.0547 | 1.9205 |
| PHL | 1991 | **0.0555** | 0.5957 | 0.0803 | 0.9566 | 0.1377 | 3.6856 | 0.0568 | 1.2362 |
| MEX | 2014 | **0.0561** | 0.6100 | 0.0701 | 0.9178 | 0.1356 | 3.4567 | 0.0577 | 1.2622 |
| CZE | 1992 | **0.0571** | 0.8168 | 0.0751 | 0.3807 | 0.0922 | 1.2300 | 0.0574 | 2.1928 |
| PER | 1994 | **0.0592** | 0.5727 | 0.0961 | 1.0000 | 0.1459 | 4.0968 | 0.0611 | 1.1995 |
| PHL | 1994 | **0.0641** | 0.5791 | 0.1006 | 0.9996 | 0.1493 | 3.9699 | 0.0654 | 1.2090 |
| CHL | 2011 | **0.0705** | 0.5922 | 0.0978 | 0.9657 | 0.1533 | 3.7434 | 0.0716 | 1.2301 |
| CHL | 1995 | **0.0712** | 0.5714 | 0.1085 | 1.0000 | 0.1578 | 4.1172 | 0.0724 | 1.1973 |
| CHL | 2013 | **0.0744** | 0.5953 | 0.0994 | 0.9573 | 0.1566 | 3.6911 | 0.0755 | 1.2354 |

This table contains the first 15 and last 15 observations of the 666 observations that are best fitted by the RGKO LC and are ordered by the size of the MDC. The variable affixed with x denotes the parameter of the function in the next column; and the variable named by a function is the standardized SSE_MDP.

**Table 10** Continued

| OMFLG | xomflg | Weibull | xweibl | Pareto | xpareto | Gupta | xgupt |
| --- | --- | --- | --- | --- | --- | --- | --- |
| 0.0775 | 0.7282 | 0.0493 | 1.2995 | 0.2027 | 2.2815 | 0.0540 | 6.0871 |
| 0.0928 | 0.7816 | 0.0509 | 1.3937 | 0.2040 | 2.1734 | 0.0512 | 5.2883 |
| 0.1519 | 1.0040 | 0.0581 | 1.8182 | 0.2057 | 1.8529 | 0.0422 | 3.4269 |
| 0.0765 | 0.7116 | 0.0527 | 1.2713 | 0.1984 | 2.3185 | 0.0586 | 6.3775 |
| 0.1337 | 0.9210 | 0.0582 | 1.6607 | 0.2030 | 1.9529 | 0.0478 | 3.9033 |
| 0.0903 | 0.7560 | 0.0549 | 1.3502 | 0.1988 | 2.2236 | 0.0572 | 5.6214 |
| 0.1562 | 1.0122 | 0.0606 | 1.8461 | 0.2036 | 1.8433 | 0.0436 | 3.3502 |
| 0.1050 | 0.8064 | 0.0569 | 1.4485 | 0.1995 | 2.1246 | 0.0546 | 4.9005 |
| 0.1429 | 0.9545 | 0.0600 | 1.7250 | 0.2023 | 1.9086 | 0.0470 | 3.6900 |
| 0.1321 | 0.9089 | 0.0593 | 1.6418 | 0.2015 | 1.9676 | 0.0496 | 3.9690 |
| 0.1502 | 0.9838 | 0.0607 | 1.7846 | 0.2026 | 1.8764 | 0.0459 | 3.5112 |
| 0.1541 | 0.9995 | 0.0614 | 1.8205 | 0.2024 | 1.8548 | 0.0450 | 3.4164 |
| 0.1101 | 0.8189 | 0.0589 | 1.4701 | 0.1981 | 2.1042 | 0.0558 | 4.7735 |
| 0.1295 | 0.8943 | 0.0604 | 1.6138 | 0.1997 | 1.9852 | 0.0513 | 4.0801 |
| 0.1588 | 1.0110 | 0.0631 | 1.8582 | 0.2018 | 1.8441 | 0.0464 | 3.3133 |
| 0.2886 | 1.2892 | 0.1394 | 2.4384 | 0.1312 | 1.6254 | 0.1067 | 2.4375 |
| 0.0889 | 0.5032 | 0.1230 | 0.9372 | 0.1122 | 2.9971 | 0.1472 | 14.4475 |
| 0.2907 | 1.2950 | 0.1407 | 2.4560 | 0.1300 | 1.6198 | 0.1075 | 2.4218 |
| 0.0873 | 0.4956 | 0.1237 | 0.9241 | 0.1107 | 3.0370 | 0.1487 | 15.1878 |
| 0.0972 | 0.5253 | 0.1251 | 0.9735 | 0.1122 | 2.8937 | 0.1469 | 12.7381 |
| 0.2903 | 1.2822 | 0.1424 | 2.4273 | 0.1280 | 1.6285 | 0.1099 | 2.4485 |
| 0.2925 | 1.2920 | 0.1430 | 2.4485 | 0.1276 | 1.6223 | 0.1100 | 2.4285 |
| 0.0933 | 0.5032 | 0.1275 | 0.9398 | 0.1076 | 2.9879 | 0.1513 | 14.2470 |
| 0.1064 | 0.5412 | 0.1297 | 1.0023 | 0.1094 | 2.8234 | 0.1497 | 11.5818 |
| 0.3414 | 1.5673 | 0.1479 | 3.0236 | 0.1264 | 1.4946 | 0.1046 | 2.0329 |
| 0.0788 | 0.4489 | 0.1286 | 0.8495 | 0.1004 | 3.2893 | 0.1584 | 20.8619 |
| 0.0879 | 0.4616 | 0.1341 | 0.8736 | 0.0964 | 3.2015 | 0.1622 | 18.5319 |
| 0.1052 | 0.4939 | 0.1421 | 0.9253 | 0.0921 | 3.0313 | 0.1667 | 15.0198 |
| 0.0891 | 0.4440 | 0.1403 | 0.8443 | 0.0879 | 3.3091 | 0.1703 | 21.2814 |
| 0.1117 | 0.5017 | 0.1463 | 0.9382 | 0.0887 | 2.9941 | 0.1702 | 14.3169 |

**Table 11** Grouped decile income data

| ID | Year | MPS | MIS | d1 | d2 | d3 | d4 | d5 | d6 | d7 | d8 | d9 | d10 |
| --- | --- | --- | --- | --- | --- | --- | --- | --- | --- | --- | --- | --- | --- |
| BEL | 1995 | 0.5895 | 0.3784 | 3.00 | 5.00 | 6.00 | 7.00 | 8.00 | 9.00 | 11.00 | 12.00 | 14.00 | 23.00 |
| BGR | 2006 | 0.5926 | 0.3725 | 2.80 | 4.70 | 5.90 | 7.10 | 8.10 | 9.40 | 10.80 | 12.40 | 14.90 | 23.90 |
| BLR | 1998 | 0.6229 | 0.4505 | 4.35 | 6.04 | 6.94 | 7.67 | 8.48 | 9.30 | 10.32 | 11.62 | 13.82 | 21.44 |
| CHL | 2011 | 0.7393 | 0.3605 | 1.60 | 2.80 | 3.60 | 4.40 | 5.40 | 6.40 | 8.00 | 10.40 | 15.20 | 42.20 |
| CHL | 1995 | 0.7523 | 0.3483 | 1.46 | 2.39 | 3.18 | 4.02 | 5.00 | 6.20 | 7.71 | 9.98 | 14.69 | 45.37 |
| CHL | 2013 | 0.7400 | 0.3653 | 1.60 | 2.80 | 3.60 | 4.60 | 5.40 | 6.60 | 8.00 | 10.40 | 15.20 | 41.80 |
| CZE | 2010 | 0.6247 | 0.4514 | 4.10 | 6.10 | 7.00 | 7.70 | 8.50 | 9.30 | 10.30 | 11.60 | 13.60 | 21.80 |
| CZE | 2008 | 0.6245 | 0.4522 | 4.20 | 6.10 | 7.00 | 7.70 | 8.50 | 9.30 | 10.30 | 11.60 | 13.70 | 21.60 |
| CZE | 1992 | 0.6111 | 0.4696 | 5.11 | 6.58 | 7.47 | 8.17 | 8.88 | 9.65 | 10.24 | 11.33 | 13.02 | 19.57 |
| DOM | 2004 | 0.7192 | 0.3547 | 1.60 | 2.70 | 3.70 | 4.60 | 5.60 | 6.90 | 8.50 | 10.90 | 15.40 | 40.10 |
| EST | 2015 | 0.6081 | 0.3597 | 2.30 | 4.40 | 5.40 | 6.40 | 7.60 | 9.10 | 10.70 | 12.70 | 15.90 | 25.60 |
| FRA | 1981 | 0.5961 | 0.3705 | 2.92 | 4.76 | 5.88 | 6.65 | 7.97 | 9.31 | 10.81 | 12.75 | 15.37 | 23.60 |
| GRC | 2015 | 0.6015 | 0.3625 | 1.90 | 4.30 | 5.70 | 6.90 | 8.00 | 9.30 | 10.70 | 12.50 | 15.40 | 25.30 |
| GRC | 2014 | 0.6032 | 0.3625 | 2.20 | 4.20 | 5.60 | 6.70 | 7.90 | 9.30 | 10.60 | 12.40 | 15.40 | 25.60 |
| HRV | 2010 | 0.5925 | 0.3695 | 2.50 | 4.50 | 5.80 | 7.10 | 8.30 | 9.50 | 10.80 | 12.80 | 15.40 | 23.30 |
| ISL | 2005 | 0.6217 | 0.4500 | 4.00 | 6.10 | 7.00 | 7.80 | 8.60 | 9.40 | 10.30 | 11.50 | 13.40 | 22.00 |
| ISR | 2007 | 0.6262 | 0.3315 | 1.82 | 3.13 | 4.27 | 5.70 | 7.03 | 8.65 | 10.48 | 12.87 | 16.59 | 29.47 |
| ISR | 2005 | 0.6316 | 0.3314 | 1.77 | 3.08 | 4.26 | 5.61 | 6.89 | 8.48 | 10.40 | 12.84 | 16.59 | 30.08 |
| ITA | 2014 | 0.5955 | 0.3704 | 2.10 | 4.70 | 6.00 | 7.10 | 8.20 | 9.40 | 10.80 | 12.40 | 14.90 | 24.40 |
| LTU | 2011 | 0.5999 | 0.3659 | 2.30 | 4.50 | 5.70 | 6.80 | 8.00 | 9.30 | 10.80 | 12.70 | 15.70 | 24.20 |
| LVA | 2008 | 0.6173 | 0.3481 | 2.20 | 3.70 | 4.80 | 6.10 | 7.40 | 8.90 | 10.60 | 12.70 | 15.60 | 28.00 |
| LVA | 2009 | 0.6163 | 0.3515 | 2.10 | 3.70 | 4.90 | 6.20 | 7.60 | 9.00 | 10.60 | 12.70 | 15.50 | 27.60 |
| MEX | 2014 | 0.7167 | 0.3625 | 1.60 | 2.90 | 3.90 | 4.80 | 5.80 | 6.90 | 8.50 | 10.80 | 15.20 | 39.00 |
| PER | 1994 | 0.7411 | 0.3425 | 1.22 | 2.25 | 3.10 | 4.06 | 5.11 | 6.44 | 7.98 | 10.37 | 14.69 | 44.78 |
| PHL | 1991 | 0.7264 | 0.3521 | 1.78 | 2.76 | 3.54 | 4.38 | 5.35 | 6.60 | 8.25 | 10.68 | 14.99 | 41.68 |
| PHL | 1994 | 0.7423 | 0.3482 | 1.66 | 2.55 | 3.31 | 4.12 | 5.08 | 6.26 | 7.83 | 10.23 | 14.47 | 44.47 |
| PRY | 2014 | 0.7221 | 0.3500 | 1.31 | 2.54 | 3.53 | 4.54 | 5.58 | 6.85 | 8.50 | 10.69 | 14.88 | 41.58 |
| PRY | 2007 | 0.7244 | 0.3490 | 1.10 | 2.40 | 3.60 | 4.50 | 5.60 | 6.90 | 8.40 | 10.70 | 14.80 | 41.90 |
| SGP | 2011 | 0.6244 | 0.3400 | 1.64 | 3.26 | 4.60 | 6.02 | 7.31 | 8.77 | 10.50 | 12.79 | 16.13 | 28.97 |
| USA | 1994 | 0.6183 | 0.3397 | 1.66 | 3.43 | 4.77 | 6.06 | 7.39 | 8.88 | 10.60 | 12.90 | 16.55 | 27.77 |

This table contains the 30 observations for the estimations in Table 9 and 10. ID denotes the three-letter country code. The column variables affixed with d denote decile income shares.
