# Supplementary material for: The first moment of income density functions and estimation of single-parametric Lorenz curves
Source: PLoS One. 2022 Jun 24;17(6):e0267828. doi: 10.1371/journal.pone.0267828 (PMC9231794; doi:10.1371/journal.pone.0267828)
Supplement: Attachment — Submitted filename: The First Moment of Income Density PLOS-ONE.pdf [file pone.0267828.s007.pdf]

<u>Review Report: PONE-D-21-23351</u>

Topic: **The First Moment of Income Density Functions and Estimation of Single-Parametric Lorenz Curves**

The first moment, the mean income point, of the income density function, and the single-parametric Lorenz curve estimates are discussed in this study. The flexibility of a parametric Lorenz function can be seen in the border of the mean income point. Using a large panel data set, the authors found the optimum parametric Lorenz function by minimizing the sum of squared errors in fitting both grouped income data and the mean income point. A zero- and unit-modal single-parametric Lorenz function is identified to be the best of eight typical optional functions in fitting most observations of a large panel data set. A Monte Carlo simulation is also performed as a robustness check of the empirical estimation. Following are my specific comments regarding the manuscript:

- Since there is only one author, it will be more appropriate to imply the ***First person narrative (singular)*** throughout the manuscript.

- The basic functions such as Pietra ratio, MPS, MDCs etc should be defined at the initial stage of the article.

- Like Fisk and lognormal Lorenz function (9 & 10), Weibull and Pareto (7 & 8) must also be defined clearly in order to maintain uniformity in defining the basic terminologies and functional forms.

- Why didn't the author consider the <u>Hierarchical Families of Lorenz Curves*</u> which perform much better than Kakwani Lorenz function and gives a robust performance in fitting actual income data across countries?
  [Reference: An Exponential Family of Lorenz Curves Author(s): José-María Sarabia, Enrique Castillo and Daniel J. Slottje Source: Southern Economic Journal, Vol. 67, No. 3 (Jan., 2001), pp. 748-756 **]**

- <u>Gastwirth's (1972) Gini bounds**</u> are non-parametric constraints that should be satisfied by the Gini index of any parametric family of Lorenz curves. Can it be concluded from the present study that Gini indices satisfy Gastwirth's bounds, since Gini coefficient is positively correlated with the MPS and negatively correlated with the MIS in the data?
  [Reference: Gastwirth,Joseph L . 1972. The estimation of the Lorenz curve and Gini index. Review of Economics and Statistics 54 : 306-16.**]**

- It is strongly suggested to summarize the main findings in section 4 & 5 in the form of single structured sentence instead in a paragraph. The current representation of findings is all very confusing.

- It would be interesting to report the current results in comparison to bi-parametric families.

- The author concluded, *"The single-parametric RGKO function outperforms the other optional single-parametric functions in the empirical estimation of group data."* Can similar conclusion be drawn to Micro data?
