# Supplementary material for: The first moment of income density functions and estimation of single-parametric Lorenz curves
Source: PLoS One. 2022 Jun 24;17(6):e0267828. doi: 10.1371/journal.pone.0267828 (PMC9231794; doi:10.1371/journal.pone.0267828)
Supplement: Attachment — Submitted filename: PlosOne Answers.pdf [file pone.0267828.s008.pdf]

Dear editor and reviewers,

I am delighted to have the chance to revise my manuscript "The first moment of income density functions and estimation of single-parametric Lorenz curves".

I highly appreciate your comments and suggestions, to which I reply one by one in the following responses (highlighted in yellow). As will become clear, I have revised the manuscript substantially in line with your comments (the revisions are highlighted in yellow as well in the manuscript) and also corrected many typos and grammatical errors.

Liang Frank Shao

• Since there is only one author, it will be more appropriate to imply the First person narrative (singular) throughout the manuscript.

Ans*: Thanks for the suggestion. I have done the revision throughout the manuscript.*

• The basic functions such as **Pietra ratio, MPS, MDC**s etc. should be defined at the initial stage of the article.

Ans*: Thanks for the suggestion. Yes, these concepts have been defined at the initial stage of the paper. For instance, MPS, MIS and MDC are defined at page 3, and then the Pietra ratio is defined at page 4 in the introduction.*

• Like **Fisk and lognormal** Lorenz function (9 & 10), **Weibull and Pareto** (7 & 8) must also be defined clearly in order to maintain uniformity in defining the basic terminologies and functional forms.

Ans: *Thank you for the suggestion. The two cumulative distribution functions have been added ahead of the definition of the two Lorenz functions.*

• It is strongly suggested to **summarize the main findings** in Section 4 & 5 in the form of **single structured sentence** instead in a paragraph. The current representation of findings is all very confusing.

Ans: *Thank you for the suggestion. I have moved the last paragraph of section 4 into Section 6 and concluded this section in one sentence as follows:*

"In summary, a single-parametric Lorenz function outperforms other options in the least squares estimation if the function's first moments match the data's first moments better than others do."

For Section 5, I have added the following concluding sentence at the end of the section:

"In summary, the flexibility of parametric LC functions in fitting a dataset can be

explained by the size of the standard deviation of their first moments in the simulation."

• Why didn't the author consider the **Hierarchical Families of Lorenz Curves*** which perform much better than Kakwani Lorenz function and gives a robust performance in fitting actual income data across countries?
[Reference: An Exponential Family of Lorenz Curves. Author(s): José-María Sarabia, Enrique Castillo and Daniel J. Slottje Source: Southern Economic Journal, Vol. 67, No. 3 (Jan., 2001), pp. 748-756]

Ans: *I did not do so because this paper focuses on single parametric functions to explain the necessity of considering the first moments of the Lorenz curves in the estimation of grouped data, whereas hierarchical families of Lorenz curves are multiple parametric functions generated by the initial exponential function. Of course, it is possible that some hierarchical families of LCs may perform much better than single parametric functions; however, the idea still holds that the first moments must be considered too for multiple parametric functions because the estimator of least squares on grouped data may not capture the first moments of the data, since the first moments of grouped data are often not explicitly included in the minimization problem regardless of what functions are used.*

The best function may change if we either consider multiparametric functions, which might be more flexible than single parametric functions in the estimation, or look into different datasets.

• Gastwirth's (1972) Gini bounds** are non-parametric constraints that should be satisfied by the Gini index of any parametric family of Lorenz curves. Can it be concluded from the present study that Gini indices satisfy Gastwirth's bounds, since Gini coefficient is positively correlated with the MPS and negatively correlated with the MIS in the data? [Reference: Gastwirth,Joseph L . 1972. The estimation of the Lorenz curve and Gini index. Review of Economics and Statistics 54 : 306-16.]

Ans: *Yes, we can conclude that the estimated Gini coefficient satisfies Gastwirth's (1972) Gini bounds since it is positively correlated with MPS and negatively correlated with MIS, and MPS and MIS are also nonparametrically bounded, which can be defined in the same way as Gastwirth's (1972) Gini bounds. I have added the following explanation to the introduction section.*

As noted in Sarabia, Castillo and Slottje (page 752, 2001), the estimator of least squares with parametric functions on grouped data does not guarantee the estimated Gini to satisfy Gastwirth's (1972) Gini bounds because the estimator may not lead to the best parametric function that captures the data's moments including the Gini coefficient; however, once the data's MIS and MPS have been well captured in the estimation then the estimated Gini will meet Gastwirth's (1972) bounds. Therefore, including the constraints of the MPS and MIS in the estimation of least squares is a stricter constraint than Gastwirth's Gini bounds.

• It would be interesting to report the current results in comparison to **biparametric families**.

Ans: *Yes, it would be interesting indeed to do the comparison. However, I would like to leave it for future work for two reasons. One is that the main result holds regardless of what parametric functions we use; that is, the least-square estimator on grouped data may not capture the first moments of the data regardless of the parametric functions to be used. The other is that there are many biparametric functions that demand much more work in identifying the best one for fitting the grouped data and hitting the first moments than working with single parametric functions, and this study focuses only on single parametric LCs. Nonetheless, I have added the following explanation at the end of the Chotikapanich function section on page 11.*

*For the **hierarchy of exponential Lorenz functions** (Sarabia, Castillo and Slottje, 2001),* $L_1(p;k,\alpha)$*, the maximum of its MDC is less than that of Chotikapanich LC; hence it does not outperform RGKO, Pareto and Kakwani functions in fitting the Lorenz curves that have an MDC equal or larger than one. The exponential Lorenz function,* $L_1(p;k,\alpha)$*, does not increase the MPS, MIS and MDC of the initial exponential function (* $\frac{e^{\sigma p}-1}{e^{\sigma}-1}$ *), because both the exponential function and Chotikapanich Lorenz function are convex, and their product shifts toward the left of the two functions. However, the other two hierarchies of exponential LCs,* $L_2(p;k,\gamma)$ *and* $L_3(p;k,\alpha,\gamma)$*, could be more flexible than* $L_1(p;k,\alpha)$ *in fitting particular data, which are beyond the task of this study.*

• The author concluded, “The single-parametric **RGKO function** outperforms the other optional single-parametric functions in the empirical estimation of grouped data.” **Can similar conclusion be drawn to Micro data?**

Ans: *Yes. The same conclusion must exist for micro data because both grouped data and micro data express the same distribution, and the first moments of the two datasets correspond to the same mean value of the distribution. However, we do not need to repeat the procedure with the density functions on micro data because each Lorenz function uniquely corresponds to its distribution density function, as do the grouped data to its micro data. Meanwhile, both the empirical exercise and simulation show that the RGKO function can catch the first moments of the data better than other functions do; my simulation shows that the excellent performance of the RGKO function is determined by the largest variation of its first moments among the optional functions, which correspondingly implies its excellent performance on micro data.*

Actually, the estimator of least squares in fitting a grouped dataset and the corresponding micro data may not lead to the same parametric function if none of the optional functions can catch the data’s first moments, because the least squares estimator could hit the mean point of micro data much better than the first moments of

grouped data, which is the key difference in the estimation with the two forms of the data; however, if there is a parametric LC function that is so flexible that it catches the first moments of grouped data, then this function will be the one to be chosen simultaneously in the estimation with both grouped and micro data. Meanwhile, many parametric Lorenz functions do not have close solutions for density functions, and it may take a huge volume to compare the estimation results of density functions with micro data.
